# Prolonged Walking with a Wearable System Providing Intelligent Auditory Input in People with Parkinson’s Disease

**DOI:** 10.3389/fneur.2017.00128

**Published:** 2017-04-06

**Authors:** Pieter Ginis, Elke Heremans, Alberto Ferrari, Kim Dockx, Colleen G. Canning, Alice Nieuwboer

**Affiliations:** ^1^Neuromotor Rehabilitation Research Group, Department of Rehabilitation Sciences, KU Leuven, Leuven, Belgium; ^2^Department of Electrical, Electronic and Information Engineering – Guglielmo Marconi, University of Bologna, Bologna, Italy; ^3^Faculty of Health Sciences, University of Sydney, Sydney, NSW, Australia

**Keywords:** Parkinson’s disease, gait, fatigue, auditory cue, attentional strategy, verbal feedback, wearable sensors

## Abstract

Rhythmic auditory cueing is a well-accepted tool for gait rehabilitation in Parkinson’s disease (PD), which can now be applied in a performance-adapted fashion due to technological advance. This study investigated the immediate differences on gait during a prolonged, 30 min, walk with performance-adapted (intelligent) auditory cueing and verbal feedback provided by a wearable sensor-based system as alternatives for traditional cueing. Additionally, potential effects on self-perceived fatigue were assessed. Twenty-eight people with PD and 13 age-matched healthy elderly (HE) performed four 30 min walks with a wearable cue and feedback system. In randomized order, participants received: (1) continuous auditory cueing; (2) intelligent cueing (10 metronome beats triggered by a deviating walking rhythm); (3) intelligent feedback (verbal instructions triggered by a deviating walking rhythm); and (4) no external input. Fatigue was self-scored at rest and after walking during each session. The results showed that while HE were able to maintain cadence for 30 min during all conditions, cadence in PD significantly declined without input. With continuous cueing and intelligent feedback people with PD were able to maintain cadence (*p* = 0.04), although they were more physically fatigued than HE. Furthermore, cadence deviated significantly more in people with PD than in HE without input and particularly with intelligent feedback (both: *p* = 0.04). In PD, continuous and intelligent cueing induced significantly less deviations of cadence (*p* = 0.006). Altogether, this suggests that intelligent cueing is a suitable alternative for the continuous mode during prolonged walking in PD, as it induced similar effects on gait without generating levels of fatigue beyond that of HE.

## Introduction

Continuous rhythmical auditory cueing (ConCue) is a well-accepted tool to improve gait in people with Parkinson’s disease (PD). Several reviews reported the immediate (1, 2) and long-term training (3, 4) effects of ConCues on spatiotemporal gait outcomes such as improved cadence, gait speed and step length, and a reduction in gait variability. However, auditory cueing was mainly studied during short-term gait trials in a laboratory setting (5). Furthermore, some side effects of ConCue have been identified. People with PD demonstrated cue dependency, expressed as a movement decline after cue removal (6–8). In addition, walking with ConCues required more metabolic energy and may thus be more fatiguing than walking without cues in both PD and healthy elderly (HE) (9, 10). Fatigue is a prevalent disabling non-motor symptom in PD (11). The mechanisms of fatigue are ill understood, but it has been associated with gait problems (12) and may have an impact on rehabilitation (13). Attentional strategies by means of verbal instructions were proposed as an alternative method for cues and were shown to have similar short-term effects (14–16). A potential drawback of verbal instruction might be that it requires more attention and translation into action than cueing and therefore increases performance variability and fatigue (17).

With the emergence of wearable technology, alternatives to ConCues are now possible. Espay et al. tested a performance-based system, which in real-time tracked the walking rhythm and modulated cueing accordingly (18). Cues no longer served as a reference target but rather as feedback on the produced stepping rhythm, requiring the person to detect and respond to the rhythm change. Although an overall cadence improvement was found after 2 weeks, results were equivocal, possibly due to the fact that abnormal cadence was supported instead of corrected by the system. More recently, a novel wearable approach was proposed, which also provided performance-based feedback, but in an intelligent rather than a continuous way (19). This system first recorded the personal optimal gait parameters, registered during a 1 min reference walk. If the selected gait parameter deviated from the individual’s optimum, corrective verbal feedback was provided through headphones. Effectiveness of this intelligent wearable system was shown following 6 weeks of at-home training, and results were retained after 4 weeks follow-up (20). Not only feedback but also auditory cueing can be provided intelligently (IntCue). It is presently unclear whether delivering IntCue is as effective as providing verbal feedback (IntFB) whereby the required stepping decrement or increment is made explicit by verbal messages such as “speed up” or “slow down.” In summary, the effects of personalized alternatives for standard cue provision need to be further investigated, including their potential effects on exertion and fatigue.

The European evidence-based guideline for physiotherapy recommends that people with PD should have daily walks of 30 min (21). Therefore, the central research question of this study was to compare the immediate effects of different cueing and feedback strategies (ConCue, IntCue, and IntFB) during a 30 min walk in people with PD. Additionally, the potential effects of these external input strategies on physical fatigue were assessed. Given the novelty of investigating a prolonged walk, a control condition without cueing or feedback and an HE control group were included. First, we hypothesized that, in contrast to HE, people with PD would have more gait difficulties during prolonged walking. We also expected that IntCue and IntFB would be as or even more effective than ConCue, as we presumed that fatigue would be reduced and that the intermittent nature of the intelligent input would be extra stimulating. Finally, we assumed that 30 min walking would induce more physical fatigue in PD compared to HE.

## Materials and Methods

### Participants

People with PD were randomly recruited from the Movement Disorders clinic of the University Hospitals Leuven based on the following inclusion criteria: (1) idiopathic PD, diagnosed according to the UK Brain Bank criteria; (2) Hoehn and Yahr stage I–III, and (3) stable PD medication for the past month and anticipated to remain so for the following 2 months. Exclusion criteria were: (1) cognitive impairment (Mini Mental State Examination score <24); (2) subjectively unable to walk unassisted for 30 min; (3) fluctuating response to levodopa, which would interfere with 30 min gait tests; (4) musculoskeletal or neurological conditions other than PD affecting gait; (5) severe hearing problems precluding headphone use for auditory information. All people with PD were tested in their subjective ON-state of the PD medication, on average 1 h after intake. HE were age matched and recruited from a database of voluntary study participants.

### Protocol

Participants performed four walks spread over a period of 6 weeks with at least 1 week interval between walks. All walks were performed in the same hall at the same time and day of the week to minimize the effects of time and PD medication. Demographic information and clinical tests were collected systematically over the four sessions prior to commencing the 30 min walk. Participant demographics, Multidimensional Fatigue Inventory (MFI) (22, 23), LASA Physical Activity Questionnaire (LAPAQ) (24), and Walk-12G (25) were collected at session 1. The Movement Disorders Society Unified Parkinson’s Disease Rating Scale—Motor Part (MDS-UPDRS III) (26) was rated at session 2. The Montreal Cognitive Assessment (MoCA) (27) was collected at session 3, and the Scale for Outcomes in Parkinson’s disease-Cognition (SCOPA-Cog) (28) was completed at session 4. After collecting demographics and clinical information, participants were provided with 5 min of rest after which they self-scored physical fatigue on a 10 cm long visual analog scale, ranging from “No fatigue” to “Maximal fatigue.”

**Figure 1:**
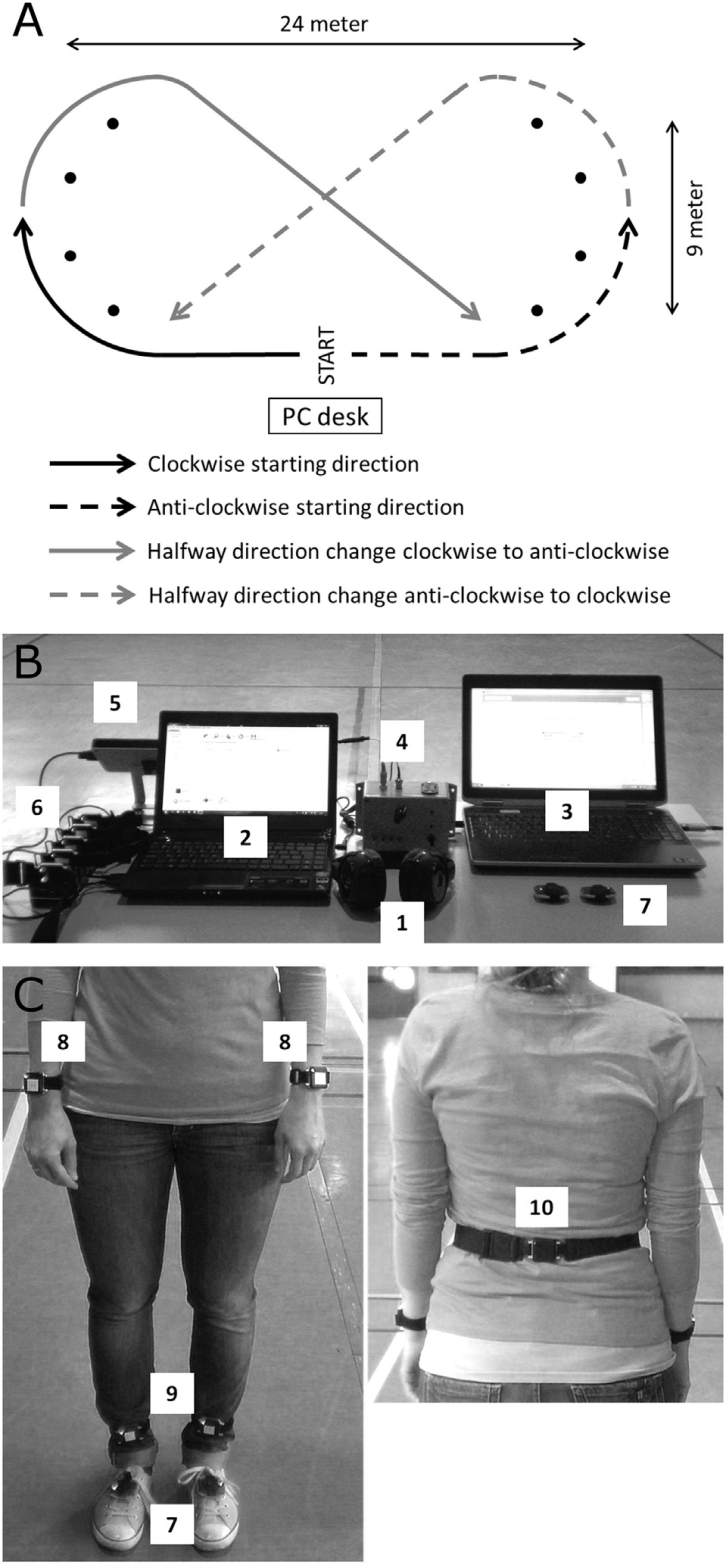
**(A)** The walking trajectory including its dimensions, randomized starting directions, and halfway direction changes. **(B)** The computerized setup with the (1) wearable headphone; (2) mobility lab computer; (3) computer with the custom MATLAB program providing the external auditory information; (4) sound synchronization box to APDM sensor system; (5) APDM antenna; (6) OPAL wearable inertial measurement units (IMUs) in their docking station; (7) EXLs1 foot-mounted wearable IMUs. **(C)** The (7) EXLs1 IMUs placed on the feet and the placement of the OPAL IMUs at the (8) wrists, (9) ankles, and (10) lower back.

Before every 30 min walk, participants performed a 1 min comfortable reference walk along a 24 m × 9 m elliptical walking trajectory (Figure 1A) comprising of wide curves to minimize the turning impact. Reference walks were recorded by two foot-mounted inertial measurement units (IMUs, EXLs1, EXEL srl, Italy), and the mean cadence was used as the reference for the subsequent 30 min walk. Each 30 min walk was performed while wearing headphones (Sennheiser RS160, Sennheiser, Germany) and in one of the following four conditions offered in a randomized order: (i) continuous cueing (ConCue), (ii) intelligent cueing (IntCue), (iii) intelligent feedback (IntFB), and (iv) no information (NoInfo). During ConCue, participants received an auditory rhythm generated by an adaptive cueing system (see Materials and Outcome Measures). The auditory rhythm was set at the mean cadence of the reference walk. Participants were instructed to follow the rhythm by stepping to the beat. During IntCue, participants received an auditory rhythm consisting of 10 beats upon real-time detection of a cadence deviation from the reference. A deviation was defined as when the mean cadence of five consecutive left and right strides deviated more than 5%. These settings were based on prior pilot work. During IntFB, participants received verbal feedback to “speed up” or “slow down” upon the real-time detection of a cadence deviation using the same criteria as in IntCue. Verbal messages were prerecorded in the local language (Dutch). During NoInfo, no external information was given during the entire walk.

Participants started their 30 min walk either in clock- or anticlockwise direction, whereby the direction was kept identical per participant over the four sessions but was randomized between persons. To counterbalance the possible effect of disease dominance, participants had to cross the walking trajectory diagonally after walking 15 min and continue the rest of the walk in the opposite direction (Figure 1A). Immediately after the walk, participants scored physical fatigue as well as their subjective rating of perceived exertion on a 6–20 Borg scale (29).

### Materials and Outcome Measures

Two foot-mounted IMUs containing a triaxial accelerometer, gyroscope, and magnetometer were attached on top of the shoes using Velcro straps. Raw angular velocities were sampled at 100 Hz and wirelessly streamed using Bluetooth to a computer (Figure 1B). A custom MATLAB (MathWorks Inc., USA) software application processed the raw data in real-time computing cadence and its deviations from the prerecorded reference during the 30 min. The algorithm to obtain cadence from the feet raw data was described elsewhere (19) and validated for PD (30). During all conditions, deviations were detected in real-time and stored for data analyses. Spatiotemporal gait variables were measured with the Mobility Lab OPAL system (APDM, USA) consisting of five IMUs attached at the participant’s wrists, ankles, and lumbar region using elastic Velcro straps (Figure 1C). The auditory information was delivered through wearable headphones.

Cadence was the primary outcome as cueing and feedback conditions targeted this gait parameter. Secondary spatiotemporal outcomes were stride length, double support time, arm swing range of motion, stride length asymmetry, and variability of cadence expressed as the coefficient of variability (%CV = SD/average × 100). These spatiotemporal gait variables were selected based on their significance for representing gait in PD (31, 32). Physical fatigue and perceived exertion were assessed as secondary outcomes using the abovementioned visual analog and Borg scale.

### Statistical Analysis

After checking data normality and homogeneity, independent *T*-tests identified differences between people with PD and HE for normally distributed descriptive data. Non-normally distributed data were analyzed using Mann–Whitney *U* tests and chi-square statistics for frequency data. Intraclass correlation coefficients (ICC_3,4_) were used to assess the agreement between the four 1-min reference walks.

The 30 min walks were split into six blocks of 5 min, and both averages and coefficients of variability of each variable per time block were calculated. A 2 group by 4 condition by 6 time-block ANOVA was used to investigate differences in spatiotemporal outcomes between groups and conditions over time, using Bonferroni corrected *post hoc* testing. Additionally, physical fatigue scores at rest and their change scores (after 30 min walking—rest) as well as Borg scores were analyzed in each condition between groups by Mann–Whitney *U* tests and in each group between conditions using Friedman tests. In case of significant Friedman tests, *post hoc* analyses were performed using Bonferroni corrected Wilcoxon Rank tests.

“Deviators” were defined as participants who deviated at least once from their reference cadence per walk. The proportion of deviators was compared in each condition and between groups using chi-square statistics. For condition differences per group, Cochran’s *Q* tests with Bonferroni corrected *post hoc* analyses were applied. The number of deviations was compared in deviators only using non-parametric Mann–Whitney *U* and Friedman tests. SPSS version 23 (IBM, USA) was used for all statistical analyses with α = 0.05.

## Results

Thirty-one people with PD and 14 age-matched HE participated in this study. All participants could perform the 30 min prolonged walk without interruptions. Data of one HE and one PD participant were excluded due to technical malfunctioning, which induced incorrect cue rhythms during their IntCue session. In addition, two PD participants were excluded because cadence deviated from the reference more than 95% of the time during ConCue, reflecting a complete inability to match the cued rhythm. Cadence of one of these participants was systematically higher than the cue rhythm, while it was systematically lower in the other. Interestingly, during IntCue, IntFB, and NoInfo both participants’ cadence did not deviate at all. Further analysis revealed that both persons were only mildly affected by the disease (H&Y I and II; LEDD 360 mg/day).

Both groups were well matched for age, body height, body weight, cognitive ability (MoCA), total self-reported daily physical activity (LAPAQ Total), and self-reported daily walking time (LAPAQ Walking) (Table 1). The test–retest agreement between the four reference minutes for cadence was excellent [ICC_3,4_ of 0.98 (95% CI 0.95–0.99)] indicating that condition effects were not confounded by reference walk instability (see Table 1 in Supplementary Material for all ICC values).

**Table 1:** **Participant characteristics**.

	Parkinson (*N* = 28)	Healthy elderly (*N* = 13)	Significance
Age (years)	62.04 (6.91)	60.23 (6.07)	*p* = 0.42
Gender (M/F)^b^	23/5	7/6	*p* = 0.07
Body weight (kg)	82.73 (15.83)	74.39 (14.63)	*p* = 0.12
Body height (cm)	174.00 (8.37)	169.85 (7.99)	*p* = 0.14
Leg length left (cm)	92.54 (5.99)	90.15 (4.20)	*p* = 0.21
Leg length right (cm)	92.14 (5.77)	90.46 (4.35)	*p* = 0.36
Disease duration (years)	10.57 (6.71)	/	/
Hoehn and Yahr (1/2/2.5/3)	1/12/7/7	/	/
MDS-UPDRS III (0–132)	34.57 (14.37)	/	/
LEDD (mg/24 h)	517.42 (312.97)	/	/
MoCA (0–30)	26.36 (2.18)	27.46 (2.22)	*p* = 0.14
Scale for Outcomes in Parkinson’s disease-Cognition (0–42)^a^	29.50 (26.00–31.25)	34.00 (32.00–35.00)	*****p*** = 0.001**
Multidimensional Fatigue Inventory (MFI) general fatigue (4–20)^a^	13.00 (9.75–15.00)	4.00 (4.00–7.00)	*****p*** < 0.001**
MFI physical fatigue (4–20)^a^	12.00 (8.75–14.25)	6.00 (5.00–8.00)	*****p*** < 0.001**
MFI reduced activity (4–20)^a^	11.50 (9.00–14.25)	6.00 (5.00–7.00)	*****p*** < 0.001**
MFI reduced motivation (4–20)^a^	9.00 (6.75–12.00)	4.00 (4.00–9.00)	*****p*** = 0.02**
MFI mental fatigue (4–20)^a^	11.50 (7.75–14.25)	5.00 (4.00–7.00)	*****p*** = 0.002**
LASA Physical Activity Questionnaire (LAPAQ) walking (min/day)^a^	14 (5–30)	11 (7–21)	*p* = 0.71
LAPAQ total (min/day)^a^	127 (56–198)	207 (105–326)	*p* = 0.14
12 G (0–87)^a^	9.50 (5.75–14.50)	0 (0–0)	*****p*** < 0.001**

*Results are reported as mean (SD) in case of parametric statistics and as median (quartile 1–quartile 3) in case of non-parametric statistics*.

*^a^Non-parametric statistics were applied*.

*^b^Chi-squared statistic*.

*Statistically significant differences (p < 0.05) are marked in bold*.

### Effects of Walking Condition on Spatiotemporal Outcomes

**Figure 2:**
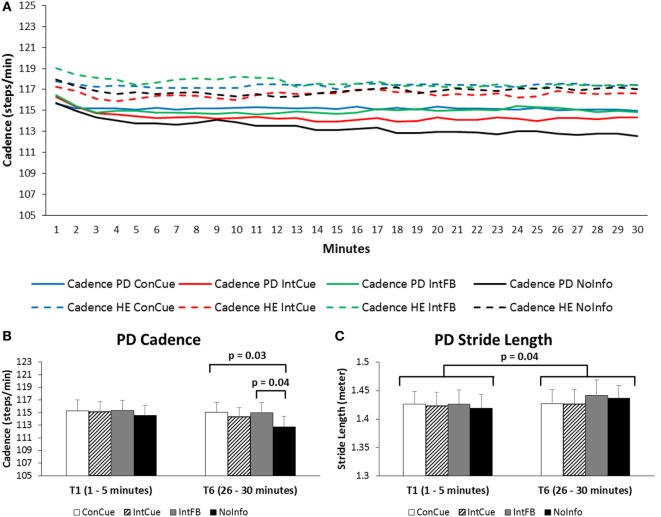
**(A)** Progression of cadence over the 30 min in the Parkinson’s disease (PD) and healthy elderly (HE) groups during the four different conditions. Full lines represent the PD group, and dotted lines represent the HE group. Averages of each minute are displayed for the full group. SDs are not displayed for reasons of clarity. See Supplementary Material Table 2 for means and SDs displayed for each 5 min interval. **(B)** Cadence of the PD group during the first and last time blocks for the four different conditions. Means and SEs (error bars) are displayed. **(C)** Stride length of the PD group during the first and sixth time blocks for the four different conditions. Means and SEs (error bars) are displayed.

Figure 2A shows the average cadence during the four conditions in both groups over the full 30 min. A group × condition × time interaction effect was found for cadence [*F*_(15,585)_ = 2.35, *p* = 0.04]. *Post hoc* analysis revealed that cadence in the PD group was significantly higher in the last three 5 min time blocks during ConCue compared to NoInfo (Δ_T4_ = 2.16 steps/min, *p* = 0.03; Δ_T5_ = 2.25 steps/min, *p* = 0.04; and Δ_T6_ = 2.34 steps/min, *p* = 0.03). Additionally, in the PD group, cadence was significantly higher in the last two 5 min time-blocks during IntFB compared to NoInfo (Δ_T5_ = 2.26 steps/min, *p* = 0.03 and Δ_T6_ = 2.29 steps/min, *p* = 0.04) (see Figure 2B). All spatiotemporal outcomes are provided in Supplementary Material Table 2.

As for the secondary spatiotemporal gait outcomes, a main time effect [*F*_(5,195)_ = 8.23, *p* = 0.001] was found for stride length, whereby the stride length during the first 5 min was significantly shorter than that of the last 5 min (Δ_T1–T6_ = −0.015 m, *p* = 0.04) (see Figure 2C). Furthermore, stride length during the second time block was significantly shorter than those during the last three time blocks (Δ_T2–T4_ = −0.010 m, *p* = 0.007; Δ_T2–T5_ = −0.011 m, *p* = 0.009; and Δ_T2–T6_ = −0.013 m, *p* = 0.02). A main time effect [*F*_(5,195)_ = 12.74, *p* < 0.001] was also found for arm range of motion, whereby range of motion during the first two time blocks was significantly smaller than during the last three time blocks (all *post hoc p*-values ≤ 0.03).

With respect to cadence CV, there was a significant group × condition interaction effect [*F*_(3,117)_ = 3.07, *p* = 0.04]. *Post hoc* analyses revealed that cadence CV was significantly higher during IntFB in PD compared to HE (Δ = 0.6%, *p* = 0.02). There was also a main time effect for cadence CV [*F*_(5,195)_ = 2.90, *p* = 0.04]. *Post hoc* tests showed an increased cadence CV during the fourth time block compared to the second time block (Δ_T2–T4_: *p* = 0.01). No significant results were found for double support time, stride length asymmetry.

### Effects of Walking Condition on Deviations

There was no significant difference between groups in the proportion of deviators in any of the four conditions. Only in the HE group, a significantly smaller proportion of deviators was seen during ConCue compared to NoInfo (*p* = 0.03) (Table 2). Among the participants who were classified as deviators, the number of deviations is presented in Table 3. There were significantly more deviations in PD compared to HE during IntFB and NoInfo (both: *p* = 0.04). Additionally, the number of deviations was smaller during ConCue and IntCue compared to NoInfo in PD (*p* = 0.006).

**Table 2:** **Number and proportion of deviators**.

Condition	Parkinson (*N* = 28)	Healthy elderly (*N* = 13)	Group effect
ConCue	15 (54%)	3 (23%)^a^	*p* = 0.10
IntCue	22 (79%)	7 (54%)	*p* = 0.15
IntFB	20 (71%)	5 (38%)	*p* = 0.08
NoInfo	21 (75%)	10 (77%)	*p* = 1.00
Condition effect	*p* = 0.08	*****p*** = 0.03**	

*Values represent the number of participants (percentage of the group) who at least once deviated from the reference*.

*^a^Significantly different from no information (NoInfo)*.

*Statistically significant differences (p < 0.05) are marked in bold*.

**Table 3:** **Number of deviations**.

	*N*	Parkinson	*N*	Healthy elderly	Group effect
ConCue	15	3.00 (2.00–8.50)^a^	3	6.00 (3.50–12.50)	*p* = 0.82
IntCue	22	5.00 (2.00–10.00)^a^	7	5.00 (1.00–17.50)	*p* = 1.00
IntFB	20	6.00 (3.75–17.00)	5	3.00 (2.00–5.00)	*****p*** = 0.04**
NoInfo	21	25.00 (8.00–83.00)	10	6.00 (4.25–18.75)	*****p*** = 0.04**
Condition effect		*****p*** = 0.006**		*p* = 0.75	

*N reports number of deviators. Number of deviations expressed as median (interquartile range) is reported per group*.

*^a^Significantly different from no information (NoInfo)*.

*Statistically significant differences (p < 0.05) are marked in bold*.

### Effects of Walking Condition on Fatigue

The results on the fatigue scores and the ratings of perceived exertion are presented in Table 4. Overall, people with PD were more physically fatigued at the beginning of the sessions than HE, which is in line with the results on the MFI listed in Table 1. Thirty minutes of walking increased physical fatigue significantly more in the PD group compared to the HE group during ConCue and IntFB conditions (*p* = 0.04 and *p* = 0.004, respectively). People with PD also rated their exertion as significantly higher than HE on the Borg scale following the 30 min of walking under all four conditions (all *p* ≤ 0.007). No significant differences between the four conditions were found within the PD or the HE groups for physical fatigue and Borg scores.

**Table 4:** **Fatigue and exertion results**.

Outcome	Condition	Parkinson	Healthy elderly	Group effect
Physical fatigue rest (0–100)	ConCue	23.0 (14.5–49.0)	4.0 (0.0–6.0)	*****p*** < 0.001**
	IntCue	21.0 (5.0–49.0)	2.0 (0.0–7.0)	*****p*** = 0.002**
	IntFB	20.5 (5.5–32.8)	1.5 (0.0–6.0)	*****p*** = 0.001**
	No information (NoInfo)	20.0 (6.5–37.0)	3.5 (0.0–5.5)	*****p*** = 0.01**
Condition effect		*p* = 0.22	*p* = 0.60	
Physical fatigue change^a^ (0–100)	ConCue	11.0 (5.5–25.0)	2.0 (1.0–10.0)	*****p*** = 0.04**
	IntCue	12.0 (7.0–26.5)	8.0 (2.0–15.0)	*p* = 0.21
	IntFB	20.0 (10.8–28.5)	3.5 (1.0–7.8)	*****p*** = 0.004**
	NoInfo	10.0 (2.5–31.5)	3.0 (1.0–10.8)	*p* = 0.11
Condition effect		*p* = 0.68	*p* = 0.14	
Borg score (6–20)	ConCue	12.5 (11.0–13.0)	8.0 (7.0–9.0)	*****p*** = 0.001**
	IntCue	12.5 (11.0–14.0)	9.0 (7.0–11.0)	*****p*** < 0.001**
	IntFB	12.5 (10.5–13.3)	9.0 (7.0–11.0)	*****p*** = 0.007**
	NoInfo	12.0 (10.0–13.0)	9.0 (7.0–11.0)	*****p*** < 0.001**
Condition effect		*p* = 0.68	*p* = 0.53	

*Values are expressed as median (interquartile range)*.

*^a^Change = immediately after 30 min walk—rest*.

*Statistically significant differences (p < 0.05) are marked in bold*.

## Discussion

This study is the first to investigate the effects of different types of auditory cueing and feedback taking into account fatigue, gait stability, and gait quality performed during an extended period of walking in PD. It was found that people with PD were able to better maintain their cadence during a continuously cued walk compared to walking without cues, especially during the last 15 min of the walk. This result illustrates that the response to cueing did not habituate with time, but quite the opposite, it helped to maintain optimal gait when fatigue or waning of attention set in. As well, the number of deviations from the reference was significantly lower during ConCue compared to NoInfo and the number of deviating participants showed a trend toward being smaller in PD. All these results are in line with previous studies showing that goal-directed motor control, elicited by means of an external reference, improved gait in PD (33). Our results extend these findings toward prolonged walking.

We presumed that intelligently provided information would be more effective than continuous cueing as the intermittent nature of the former input would avoid dependency and habituation, previously reported to occur as a result of continuous cueing (6, 7). Furthermore, as performance deviations triggered the onset of information, intelligent solutions might increase the person’s alertness avoiding dependency and habituation even more. In fact, we found a similar stabilizing effect on gait for IntCue as for ConCue, even though cues were only presented when gait deviated. Interestingly, the number of deviations was significantly reduced during IntCue whereas this was not the case for IntFB. This may indicate that the actual presence of the target even when applied intermittently is more beneficial for keeping cadence than occasional verbal information, which is less clear about how to change the walking parameters. However, in the last time blocks of the walk, people with PD were better able to increase their cadence in response to IntFB than during IntCue. Along the same lines, there was an overall time effect for stride length improvement mostly seen during IntFB. Taken together, this may indicate that the feedforward action of clear targets, as indicated by cues rather than by verbal feedback, appears more useful to preserve gait stability. In contrast, the feedback approach may be more effective to invigorate gait, maybe driven by the allocation of cognitive resources, as recalling the internal motor plan is needed more without the presence of a target. The fact that cognition and or attention are required more when processing verbal feedback rather than auditory cues is also supported by the finding that a significant increase in cadence variability was apparent in PD and not in HE during IntFB. Hausdorff and colleagues demonstrated that increased gait variability is a marker of heightened attention allocated to gait (34). Another explanation of the differential effects of IntCue and IntFB is that by providing a clear motor target (cue) a limited number of possible movement corrections are indicated. By providing a verbal instruction to speed up or slow down gait, more degrees of freedom are allowed and thus effects may be larger. This is in line with Baker et al. who also found that attentional strategies induced larger effects than auditory cues on gait parameters (14).

In contrast to our hypothesis and previous studies (9, 10), no differences were found between the walking conditions with regard to fatigue and ratings of perceived exertion. It is however noteworthy that the change scores of physical fatigue were significantly higher in PD than in HE during ConCue and IntFB. This suggests that responding to continuous cues as well as reacting to intermittent verbal feedback created an extra burden to people with PD. This pattern fits with the idea that IntFB requires more cognitive load (14). In addition, the fact that ConCue was more burdensome corresponds with the finding that continuous cues during treadmill walking induced a greater metabolic cost than non-cued gait in both PD and HE (9, 10).

Overall, we found strikingly higher fatigue and exertion levels in PD compared to HE during 30 min of walking, which is in line with earlier work (35). Fatigue is one of the most disabling non-motor symptoms in PD and generally defined as a feeling of tiredness, lack of energy, and exhaustion or as a reduced capacity to initiate or sustain voluntary activities (13, 36, 37). Although self-perceived levels of fatigue were high, the measured impact on gait quality was relatively minor as during NoInfo only cadence deteriorated with time.

It is interesting that two persons with PD were unable to follow the rhythm even though cue settings were based on individualized reference walks. This suggests that auditory cueing mechanisms are more complex than merely providing a movement target and underpins earlier findings of individual differences in cue efficacy (5, 38). The fact that two of the least affected persons with PD experienced difficulties with ConCue concurs with earlier work showing that those with more severe disease benefited most from cues (39). Together with the finding that these two persons responded well to the intelligent conditions suggests that persons with mild PD may not benefit from continuous cueing. Another explanation for an individualized response to cueing is the fact that the basal ganglia have a role in rhythm perception (38). However, recent findings point to a facilitating effect of predictive rhythmic stimulation on basal ganglia–premotor cortex interactions (40), despite a potential deficit of rhythm perception in PD (5). In addition, the type of acoustic input may also explain a differential response, the sound of feet walking over gravel was found to be more effective than metronome cues in some people with PD (41, 42).

Surprisingly, we found that stride length and arm swing increased over the 30 min period in both PD and HE. We explain this finding as a possible effect of prolonged walking, whereby the gait pattern gets “into the groove,” as also shown in inactive HE (43). Given the strong correlation between arm swing and stride amplitude (44), spontaneous overflow from one parameter to the other may also have occurred. Recent work also showed a significant increase in stride length following a repeated sit-to-stand protocol in both PD and age-matched HE (45). These effects, not demonstrated during short gait trials in gait laboratories, support the European guideline’s recommendation to undertake 30 min bouts of brisk walking as a rehabilitation strategy for PD (21).

Our results provide some new clues on how to apply cueing optimally in a rehabilitation context. To obtain positive effects on both gait quality and stability, providing cueing in an intelligent approach alternated with adaptive verbal feedback may prove to be more effective than using continuous cueing and may lead to less overload and fatigue. Given the emergence of smartphone and wearable sensing technology, self-regulating systems to provide external information intelligently are in the making (18, 19, 46), opening their use into daily routine (47). A recent pilot study showed the feasibility and effectiveness of such a wearable system during a home-based minimally supervised training period of 6 weeks in PD (20). While these tools are not widely available yet, physiotherapists can use auditory cues in smartphone apps and verbal instructions intermittently based on clinical observation of gait deterioration. Future studies should test the effectiveness of a combined approach of delivering both IntFB and IntCue, determine which cueing mode is best for different clinical profiles and identify if the benefits can be consolidated during long-term home training. A limitation of this study was the use of self-reported fatigue and exertion scales instead of objective outcomes such as metabolic cost through VO_2_ measurement. Another limitation was the significant group differences in fatigue and SCOPA-Cog scores at baseline. The MoCA scores, however, were matched between groups, suggesting that participants were equally capable of processing auditory information.

In conclusion, people with PD show greater cadence deterioration and report more fatigue and exertion during 30 min of walking than healthy age-matched controls. Intelligently applied cueing was most successful in maintaining gait stability. Intelligent feedback led to the best cadence at the end of the walk but also increased cadence variability and fatigue. Although continuous cueing was beneficial for reducing gait deviations, persons with PD reported more fatigue during this cueing mode than HE. We recommend intelligently applied cueing and possibly also adaptive feedback approaches as the most appropriate gait rehabilitation tools for people with PD when undertaking prolonged walking bouts.

## Ethics Statement

This study was carried out in accordance with the recommendations of Ethics Committee of the KU Leuven with written informed consent from all subjects. All subjects gave written informed consent in accordance with the Declaration of Helsinki. The protocol was approved by the Ethics Committee of the KU Leuven.

## Author Contributions

PG, EH, CGC, and AN designed the study; AF developed the automated cueing and feedback system; PG and KD collected, processed, and analyzed the data; PG wrote the manuscript; and EH, AF, KD, CGC, and AN revised the manuscript.

## Conflict of Interest Statement

AF has a significant financial interest in mHealth Technologies, a company that may have a commercial interest in the results of this research. All other authors declare no competing interests.
