# Supplementary material for: Prolonged Walking with a Wearable System Providing Intelligent Auditory Input in People with Parkinson’s Disease
Source: Front Neurol. 2017 Apr 6;8:128. doi: 10.3389/fneur.2017.00128 (PMC5382170; doi:10.3389/fneur.2017.00128)
Supplement: Supplementary file 1 [file Table_1.DOCX]

Supplementary Material

Prolonged Walking with a Wearable System Providing Intelligent Auditory Input in People with Parkinson’s Disease

Pieter Ginis^1^, Elke Heremans^1^, Alberto Ferrari^2^, Kim Dockx^1^, Colleen G. Canning^3^, Alice Nieuwboer^1^*

^1^Neuromotor Rehabilitation Research Group, Department of Rehabilitation Sciences, KU Leuven, Leuven, Belgium

^2^Department of Electrical, Electronic and Information Engineering – Guglielmo Marconi, University of Bologna, Bologna, Italy

^3^Faculty of Health Sciences, University of Sydney, Sydney, New South Wales, Australia

# * Correspondence: Alice Nieuwboer [alice.nieuwboer@kuleuven.be](mailto:alice.nieuwboer@kuleuven.be)

| Supplementary Table 1 – Reference minute reliability over four test sessions | | |
| --- | --- | --- |
|  | Parkinson | Healthy Elderly |
| Cadence (steps/min) | 0.98 (0.96 – 0.99) | 0.98 (0.95 – 0.99) |
| Stride length (m) | 0.98 (0.97 – 0.99) | 0.99 (0.99 – 1.00) |
| Double support time (% GCT) | 0.90 (0.82 – 0.95) | 0.72 (0.32 – 0.91) |
| Arm ROM (degrees) | 0.95 (0.91 – 0.98) | 0.96 (0.92 – 0.99) |
| Stride length asymmetry | 0.88 (0.79 – 0.94) | 0.78 (0.50 – 0.93) |
| Cadence variability (%) | 0.57 (0.22 – 0.78) | 0.70 (0.31 – 0.90) |
| Values represent ICC_(3,4)_ (95% confidence interval); GCT = Gait cycle time; ROM = Range of motion; variability is expressed as coefficient of variability | | |
