# Supplementary material for: Prolonged Walking with a Wearable System Providing Intelligent Auditory Input in People with Parkinson’s Disease
Source: Front Neurol. 2017 Apr 6;8:128. doi: 10.3389/fneur.2017.00128 (PMC5382170; doi:10.3389/fneur.2017.00128)
Supplement: Supplementary file 2 [file Table_2.DOCX]

Supplementary Material

Prolonged Walking with a Wearable System Providing Intelligent Auditory Input in People with Parkinson’s Disease

Pieter Ginis^1^, Elke Heremans^1^, Alberto Ferrari^2^, Kim Dockx^1^, Colleen G. Canning^3^, Alice Nieuwboer^1^*

^1^Neuromotor Rehabilitation Research Group, Department of Rehabilitation Sciences, KU Leuven, Leuven, Belgium

^2^Department of Electrical, Electronic and Information Engineering – Guglielmo Marconi, University of Bologna, Bologna, Italy

^3^Faculty of Health Sciences, University of Sydney, Sydney, New South Wales, Australia

# * Correspondence: Alice Nieuwboer [alice.nieuwboer@kuleuven.be](mailto:alice.nieuwboer@kuleuven.be)

| Supplementary Table 2 – Spatiotemporal outcomes | | | | | | | | | | | | |  |
| --- | --- | --- | --- | --- | --- | --- | --- | --- | --- | --- | --- | --- | --- |
|  |  | Parkinson (n=28) | | | | |  | | Healthy Elderly (n=13) | | | |  |
| Variable | Time-block | ConCue | IntCue | IntFB | NoInfo |  | | ConCue | | IntCue | IntFB | NoInfo | |
| Cadence (steps/min) | | | | | | | | | | | | | |
|  | T1 | 115.26 (9.34) | 115.09 (8.66) | 115.33 (8.81) | 114.57 (8.54) |  | | 117.44 (9.57) | | 116.45 (7.75) | 118.19 (8.63) | 117.08 (8.62) | |
|  | T2 | 115.20 (9.17) | 114.30 (8.49) | 114.75 (8.71) | 113.84 (8.99) |  | | 117.14 (9.67) | | 116.27 (7.88) | 117.96 (8.97) | 116.58 (9.29) | |
|  | T3 | 115.23 (8.95) | 114.14 (8.39) | 114.74 (8.58) | 113.37 (9.05) |  | | 117.39 (9.59) | | 116.60 (8.41) | 117.70 (8.66) | 116.52 (9.54) | |
|  | T4 | 115.22 (8.96) | 114.97 (8.35) | 115.00 (8.59) | 113.06 (9.01) |  | | 117.50 (9.51) | | 116.79 (8.56) | 117.46 (8.76) | 116.94 (9.84) | |
|  | T5 | 115.17 (8.83) | 114.14 (8.22) | 115.17 (8.55) | 112.91 (8.88) |  | | 117.37 (9.54) | | 116.44 (8.36) | 117.24 (8.76) | 117.01 (9.95) | |
|  | T6 | 115.05 (8.74) | 114.27 (8.21) | 115.00 (8.68) | 112.71 (8.97) |  | | 117.45 (9.45) | | 116.67 (8.75) | 117.41 (9.04) | 117.08 (9.91) | |
| Stride length (meter) | | | | | | | | | | | | | |
|  | T1 | 1.43 (0.12) | 1.42 (0.13) | 1.43 (0.13) | 1.42 (0.13) |  | | 1.47 (0.14) | | 1.47 (0.13) | 1.49 (0.14) | 1.48 (0.13) | |
|  | T2 | 1.43 (0.13) | 1.42 (0.14) | 1.43 (0.13) | 1.42 (0.13) |  | | 1.47 (0.14) | | 1.47 (0.13) | 1.48 (0.14) | 1.49 (0.13) | |
|  | T3 | 1.43 (0.13) | 1.42 (0.14) | 1.44 (0.14) | 1.43 (0.13) |  | | 1.47 (0.14) | | 1.48 (0.13) | 1.49 (0.14) | 1.49 (0.13) | |
|  | T4 | 1.44 (0.13) | 1.42 (0.14) | 1.44 (0.14) | 1.44 (0.13) |  | | 1.48 (0.13) | | 1.49 (0.12) | 1.49 (0.13) | 1.49 (0.12) | |
|  | T5 | 1.43 (0.13) | 1.42 (0.14) | 1.44 (0.15) | 1.44 (0.12) |  | | 1.48 (0.13) | | 1.49 (0.13) | 1.50 (0.13) | 1.49 (0.12) | |
|  | T6 | 1.43 (0.13) | 1.43 (0.14) | 1.44 (0.14) | 1.44 (0.12) |  | | 1.49 (0.13) | | 1.49 (0.13) | 1.51 (0.13) | 1.50 (0.13) | |
| Double support time (% gait cycle time) | | | | | | | | | | | | | |
|  | T1 | 19.69 (2.88) | 19.58 (2.24) | 19.37 (3.25) | 19.43 (2.77) |  | | 18.93 (2.80) | | 19.17 (2.14) | 19.35 (1.97) | 19.61 (2.76) | |
|  | T2 | 19.70 (3.05) | 19.83 (2.46) | 19.38 (3.42) | 19.59 (3.10) |  | | 18.87 (3.02) | | 19.38 (2.25) | 19.18 (2.07) | 19.91 (2.70) | |
|  | T3 | 19.51 (3.17) | 19.90 (2.75) | 19.24 (3.50) | 19.46 (3.29) |  | | 18.84 (3.34) | | 19.08 (2.31) | 19.17 (2.02) | 19.71 (3.05) | |
|  | T4 | 19.44 (3.12) | 19.73 (2.73) | 19.02 (3.42) | 19.32 (3.37) |  | | 18.86 (3.10) | | 19.39 (2.34) | 19.27 (1.89) | 19.47 (3.24) | |
|  | T5 | 19.32 (3.48) | 19.54 (2.85) | 18.90 (3.57) | 19.27 (3.43) |  | | 18.89 (3.13) | | 19.27 (2.48) | 19.17 (2.08) | 19.45 (3.51) | |
|  | T6 | 19.34 (3.60) | 19.23 (2.87) | 18.85 (3.64) | 19.25 (3.35) |  | | 18.99 (3.14) | | 19.18 (2.62) | 19.04 (2.24) | 19.33 (3.74) | |
| Arm swing range of motion (degrees) | | | | | | | | | | | | | |
|  | T1 | 21.70 (13.34) | 23.78 (15.03) | 23.65 (14.7) | 23.23 (15.55) |  | | 24.48 (10.62) | | 22.83 (9.76) | 24.45 (12.51) | 24.41 (9.20) | |
|  | T2 | 23.24 (15.34) | 23.85 (15.33) | 24.39 (14.95) | 23.69 (15.54) |  | | 26.05 (13.52) | | 23.56 (9.14) | 25.24 (13.37) | 25.01 (9.54) | |
|  | T3 | 25.93 (17.66) | 24.04 (14.64) | 25.68 (15.46) | 24.13 (15.21) |  | | 26.22 (13.92) | | 23.27 (8.74) | 25.84 (12.65) | 26.41 (9.26) | |
|  | T4 | 26.38 (17.91) | 23.98 (14.37) | 27.02 (16.79) | 24.34 (15.50) |  | | 26.51 (13.14) | | 24.92 (8.93) | 27.18 (10.57) | 27.92 (10.06) | |
|  | T5 | 26.21 (16.88) | 24.20 (15.09) | 27.29 (16.83) | 25.57 (16.33) |  | | 26.76 (13.17) | | 25.69 (8.24) | 28.06 (11.30) | 28.91 (10.57) | |
|  | T6 | 25.48 (15.81) | 24.67 (15.69) | 27.70 (17.48) | 25.24 (15.28) |  | | 27.08 (12.49) | | 26.00 (8.15) | 29.01 (12.00) | 29.47 (10.97) | |
| Stride length asymmetry (ratio) | | | | | | | | | | | | | |
|  | T1 | 1.09 (0.42) | 1.08 (0.52) | 1.05 (0.53) | 1.15 (0.55) |  | | 1.12 (0.60) | | 1.05 (0.38) | 0.90 (0.31) | 1.01 (0.46) | |
|  | T2 | 1.11 (0.43) | 1.08 (0.52) | 1.05 (0.48) | 1.18 (0.59) |  | | 1.13 (0.67) | | 1.03 (0.32) | 0.92 (0.32) | 0.97 (0.50) | |
|  | T3 | 1.15 (0.48) | 1.08 (0.51) | 1.12 (0.55) | 1.17 (0.61) |  | | 1.13 (0.61) | | 1.01 (0.32) | 0.96 (0.38) | 1.05 (0.63) | |
|  | T4 | 1.18 (0.54) | 1.11 (0.51) | 1.14 (0.56) | 1.21 (0.62) |  | | 1.14 (0.62) | | 1.02 (0.30) | 1.04 (0.36) | 1.08 (0.74) | |
|  | T5 | 1.18 (0.55) | 1.12 (0.56) | 1.17 (0.62) | 1.19 (0.63) |  | | 1.09 (0.48) | | 0.98 (0.28) | 1.00 (0.34) | 1.08 (0.73) | |
|  | T6 | 1.17 (0.55) | 1.13 (0.54) | 1.18 (0.60) | 1.22 (0.60) |  | | 1.04 (0.39) | | 1.03 (0.30) | 0.96 (0.32) | 1.06 (0.71) | |
| Cadence variability (%) | | | | | | | | | | | | | |
|  | T1 | 1.62 (0.57) | 1.84 (0.56) | 1.91 (0.72) | 2.08 (0.97) |  | | 1.76 (1.02) | | 1.51 (0.78) | 1.44 (0.76) | 1.63 (0.74) | |
|  | T2 | 1.63 (0.57) | 1.79 (0.83) | 1.81 (0.82) | 1.85 (0.94) |  | | 1.71 (1.05) | | 1.36 (0.55) | 1.26 (0.38) | 1.48 (0.77) | |
|  | T3 | 1.74 (0.66) | 1.70 (0.63) | 1.82 (0.83) | 1.86 (0.76) |  | | 1.81 (1.00) | | 1.40 (0.56) | 1.36 (0.69) | 1.55 (0.97) | |
|  | T4 | 1.81 (0.63) | 1.86 (0.8) | 1.98 (0.74) | 1.96 (0.85) |  | | 1.79 (1.04) | | 1.50 (0.42) | 1.54 (0.70) | 1.66 (1.05) | |
|  | T5 | 1.77 (0.59) | 1.74 (0.73) | 2.02 (1.06) | 1.84 (0.70) |  | | 1.75 (0.92) | | 1.46 (0.50) | 1.21 (0.35) | 1.59 (1.01) | |
|  | T6 | 1.79 (0.66) | 1.85 (0.66) | 2.03 (0.99) | 1.89 (0.66) |  | | 1.67 (0.77) | | 1.56 (0.53) | 1.22 (0.32) | 1.58 (0.99) | |
| Results reported as mean (SD). | | | | | | | | | | | | | |
